# SADI-S WITH RIGHT GASTRIC ARTERY LIGATION: TECHNICAL SYSTEMATIZATION AND EARLY RESULTS

**DOI:** 10.1590/0102-6720201600S10021

**Published:** 2016

**Authors:** Jordi Pujol GEBELLI, Amador Garcia Ruiz de GORDEJUELA, Almino Cardoso RAMOS, Mario NORA, Ana Marta PEREIRA, Josemberg Marins CAMPOS, Manoela Galvão RAMOS, Eduardo Lemos de Souza BASTOS, João Batista MARCHESINI

**Affiliations:** 1 Hospital Universitari de Bellvitge, Barcelona, Spain;; 2 Gastro-Obeso-Center Advanced Surgical Institute, São Paulo, Brazil;; 3 Centro Hospitalar Entre Douro e Vouga, Portugal;; 4 Federal University of Pernambuco, Recife, Brazil;; 5 Clínica Marchesini, Curitiba, PR, Brazil

**Keywords:** Bariatric surgery, Morbid obesity, Biliopancreatic diversion

## Abstract

**Background:**

Bariatric surgery is performed all over the world with close to 500.000 procedures per year. The most performed techniques are Roux-en-Y gastric bypass and sleeve gastrectomy. Despite this data, the most effective procedure, biliopancreatic diversion with or without duodenal switch, represents only no more than 1.5% of the procedures. Technical complexity, morbidity, mortality, and severe nutritional adverse effects related to the procedure are the main fears that prevent most universal acceptance.

**Aim:**

To explain the technical aspects and the benefits of the SADI-S with right gastric artery ligation as an effective simplification from the original duodenal switch.

**Methods:**

Were included all patients undergoing this procedure from the November 2014 to May 2016, describing and analysing aspects of this technique, the systematization and early complications associated with the procedure.

**Results:**

A series of 67 patients were operated; 46 were women (68.7%); mean age of the group was 44 years old (33-56); and an average BMI of 53.5 kg/m^2^ (50-63.5). Surgical time was 115 min (80-180). A total of five patients (7.5%) had any complication and two (2.9%) had to be reoperated. There were two patients with leak, one at the duodenal stump and other at the esophagogastric angle. There was no mortality. Patients stayed at the hospital a median of 2.5 days (1-25).

**Conclusions:**

SADI-S with right gastric artery ligation is a safe procedure with few preliminary complications. The technical variations introduced to the classical duodenal switch are reproducible and may allow this procedure to be more popular. All the complications in this series were not related to the ligation of the right gastric artery.

## RESUMO

**Racional:**

Cerca de 500.000 cirurgias bariátricas são realizadas a cada ano em todo o mundo. As técnicas mais realizadas são o bypass gástrico em Y-de-Roux e a gastrectomia vertical. A derivação biliopancreática, com ou sem duodenal switch, é considerada técnica mais eficaz no tratamento cirúrgico do paciente obeso mórbido; entretanto, representa não mais do que 1,5% dos procedimentos na atualidade, pois sua complexidade técnica, morbimortalidade e graves efeitos adversos nutricionais impedem aceitação mais universal.

**Objetivo:**

Descrever os aspectos técnicos e os benefícios do SADI-S com ligadura da artéria gástrica direita como um modo simplificado do duodenal switch original.

**Métodos:**

Foram incluídos todos os pacientes submetidos a este procedimento entre novembro 2014 e maio de 2016. Além da descrição da sistematização da técnica operatória, foram analisadas as complicações precoces associadas ao procedimento.

**Resultados:**

Uma série de 67 pacientes foi operada no período analisado; 46 eram mulheres (68,7%) e a média de idade foi de 44 anos (33-56). O IMC médio foi de 53,5 kg/m^2^ (50-63,5). O tempo cirúrgico médio foi de 115 min (80-180) e a permanência hospitalar média foi de 2,5 dias (1-25). Complicações foram observadas em cinco pacientes (7,5%) e dois (2,9%) tiveram de ser reoperados. Duas pacientes evoluíram com fístula, uma no coto duodenal e outra na junção esofagogástrica. Não houve mortalidade.

**Conclusão:**

SADI-S com ligadura da artéria gástrica direita é procedimento seguro com poucas complicações precoces. A simplificação técnica em relação ao duodenal switch clássico pode permitir que este procedimento se torne mais popular. Todas as complicações observadas nesta série não estavam relacionadas com a ligadura da artéria gástrica direita.

## INTRODUCTION

Considering the first historical cases of jejunoileal bypass done in the beginning of the 1950s by Victor Henriksson in Sweden and Richard Varco in United States of America, bariatric surgery has completed a long medical history with the proposition of several surgical alternatives, reaching global numbers around 500.000 cases yearly^2^ ^,^ ^7^ ^,^ ^9^.

Influenced by the jejunoileal bypass serious side effects involving severe nutritional problems, liver and kidney complications and poor quality of life related to this pure malabsortive cluster of procedures, the evolution of the bariatric surgery was divided in two main pathways. 

The first one, with Mason and Ito in1967 launching the first generation omega gastric bypass, that overtime resulted in the nowadays-leading Roux-en-Y gastric bypass (RYGB)^2^ ^,^ ^20^ ^,^ ^22^ ^,^ ^25^.Since the first series in 1994 by Wittgrove, the laparoscopic RYGB has been the most performed bariatric procedure in the last 22 years and was considered as the gold standard in this field^34^.For the last five years sleeve gastrectomy has emerged and gained surgeon's preference exponentially up to reaches the position of the most performed surgery in some countries, including USA^2^ ^,^ ^6^. Despite their success both procedures achieve moderate results in terms of weight loss and have a relative high 20-25% index of weight loss failure or weight regain increasing the worldwide data of revisional surgery in the recent years^6^ ^,^ ^10^ ^,^ ^15^.

The second pathway for development of bariatric surgery was based in the improvement attempts of the original jejunoileal bypass with different lengths of bowel exclusion and association with moderate gastric restriction resulting in a second generation of malabsortive operations: the classic biliopancreatic diversion proposed by Nicola Scopinaro *et al.* in 1979 ^32^. In the next years Marceau and Hess added some technical changes resulting in another type of biliopancreatic diversion: the sleeve gastrectomy with duodenal switch (DS)^12^ ^,^ ^18^. These procedures have also more than 35 years of history and have demonstrated the best results ever in terms of durable weight loss and comorbidities resolution^4^ ^,^ ^12^ ^,^ ^18^. Nevertheless in the last International Federation for Surgery of Obesity (IFSO) bariatric surgery survey they only represented 1.5% of the worldwide series^2^.

The main reasons for such conservative recommendation numbers is that both, biliopancreatic diversion and DS are usually associated with the high morbidity and mortality rates, high technical complexity and elevated long-term nutritional sequelae. However, if we carefully examine the more recent literature about DS we may find studies with large follow-up pointing for good results and few complication numbers but even though this literature is unanimous, this is the technically most demanding and complex bariatric procedure^23^ ^,^ ^28^.

Based in the excellent results of DS butlooking for some easier and safer surgical technical alternative, Sánchez-Pernaute*et al.* proposed in 2007 the single-anastomosis duodenoileal bypass with sleeve gastrectomy (SADI-S). It was described as a technical simplification of the DS to reduce its complexity, morbidity and mortality maintaining the same weight loss and comorbidities resolution results^29^.

Since 2007 several papers with worldwide series evaluated the safety, efficacy and feasibility of the SADI-S. Overall results show similar weight loss to DS with less operating time and less morbidity and mortality rates^22,29-31^.

The hardest part of the procedure is related to the duodenal approach and the duodenoileal anastomosis. The right gastric artery ligation is a technical gesture described from the Whipple's procedure with pyloric preservation. Marchesini demonstrated that it might be useful for facilitate the duodenoileal anastomosis of the DS without compromising the gastric sleeved blood supply and allowing an easier anastomosis relieving the tension between both intestinal segments^19^ ^,^ ^24^.

The aim of this paper was to report the technical aspects of surgical systematization of the SADI-S with right gastric artery ligation and the early outcomes of the procedure.

## METHODS

The records of the patients underwent to laparoscopic SADI-S with right gastric artery ligation between November 2014 and May 2016 were reviewed. The main indication for this surgery was patients with BMI over 50 kg/m^2^, especially those with severe metabolic comorbidities. Patients with contraindication for malabsortive procedures were excluded, as well as those with moderate or large hiatal hernia and complicated erosive esophagitis.

### Preoperative set-up

After adequate selection and appropriate surgical recommendation all patients were submitted to multidisciplinary team preparation. During the preoperative work up patients had also specialized evaluations depending on their comorbidities in order to have the proper control targeting a surgery in the best condition as possible. All patients were submitted to psychological and psychiatric if necessary, evaluation prior to surgery. Once the patient was considered suitable to surgery was also evaluated by anaesthesiology and was required to perform a very low calorie diet, pulmonary physiotherapy and physical education during two weeks prior to surgery. The patients were then informed about the routines for the procedure and sign the informed consent.

### Surgical procedure

#### Position of the patient and the surgical team

The patients were placed in the supine position with open legs. The table tilts changed between Trendelenburg and reverse-Trendelenburg position depending on the step of the surgery. Patient was secured to the operative table by foot tops, two belts at the height of the knees and an abdominal brace. The surgeon stood between patient's legs, except for the counting of the small bowel when changed to the left side of the patient. The first assistant remained on the left side of the patient and the second on the right side. Central venous access and urine catheters were not used. Antibiotic prophylaxis was routinely done. Prevention of thromboembolism was made with use of graduated compression stockings, intermittent pneumatic boots, enoxaparin and early walking.

#### Pneumoperitoneum and placement of the trocars

**FIGURE 1:**
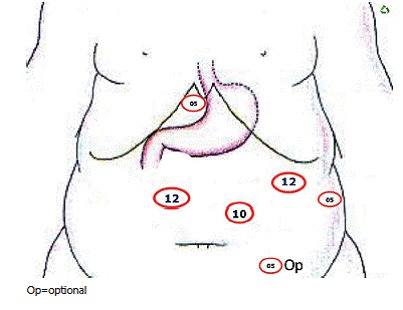
Trocars set up for SADI-S with right gastric artery ligation

Pneumoperitoneum was created by direct Veress needle puncture at the Palmer's point. A 15 mmHg CO_2_ abdominal pressure was set for all the procedure with a 5-6 trocars set up. The first trocar (10-12 mm) is placed 2-3 cm to the left of the midline 15-18 cm caudal from the xiphoid for the placement of a 10 mm/30 degrees lens. Both sides of the camera 5-10 cm away at the same line were placed two 12 mm trocars for both working hands of the surgeon. The assistant placed a 5 mm trocar very lateral in the left side of the patient (anterior axillary line) 2-3 cm from the last costal bone. Another 5 mm trocar was placed at the xiphoid to liver retraction. Finally another optional 5 mm trocar was placed close to the umbilicus in the left pararectal space to facilitate the bowel counting and mobilization (Figure 1).

#### Mobilization of the stomach

**FIGURE 2:**
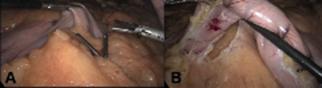
A) Starting greater curvature dissection in the distal stomach heading to the esophagogastric angle; B) antrum dissection approaching duodenal liberation

The surgery starts with the dissection of the esophagogastric angle in order to expose the left side of the crura. The greater curvature was mobilized with ultrasonic scissor from bottom to top removing all the adhesions in the lesser sac in order to have a complete dissection of the stomach (Figure 2A).

#### Duodenal dissection and right gastric artery ligation (Figure 2B)

**FIGURE 3:**
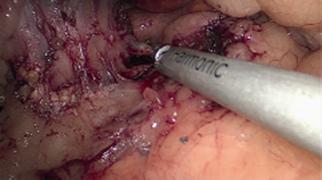
Right gastric artery ligation with harmonic energy

To make easier and prepare the stomach, pylorus and duodenum the best approach is done through the gastric greater curvature. The dissection of the first portion of the duodenum begins from the distal antrum with complete liberation of the duodenum up to the gastro-duodenal artery. The antrum is tensioned and the pyloric region is retracted upward and to the right of the patient. The assistant holds the antrum and the first portion of the duodenum to facilitate the dissection of the posterior compartment and first part of the duodenum. The posterior aspect of the duodenum is dissected until the exposure of the gastro-duodenal artery. The right gastric artery is dissected posteriorly at the level of the pylorus. It can be clipped or ligated with ultrasonic scissor (Figure 3). Special care is taken at this level with the common bile duct. The aspect of the exposure of the surgical field in order to identify the right gastric artery has the shape of a rectangle. The upper part is the stomach, the lower part is the pancreas, the left aspect is the duodenum and pylorus, and the right side is the body of the stomach. The right gastric artery will be located in the left of this rectangle.

**FIGURE 4:**
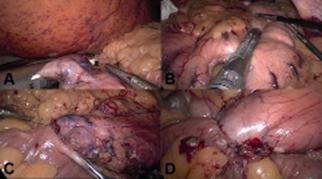
A) Duodenal transaction; B) sleeve gastrectomy staplering: sequence of firings; C) duodenoileal anastomosis first row; D) final aspect

Then the duodenum can be safely sectioned with a blue or white cartridge (Figure 4A). Considering the thickness and the vascularization of the duodenum we prefer a white one. This technical gesture allows a wide mobilization of the proximal ending of the duodenum and stomach without compromising the blood supply.

Now the stomach with first portion of duodenum can be orientated to a medial position and in general the tip of the proximal duodenum can reach the infra-mesocolic abdomen.

#### Sleeve gastrectomy

With the stomach fully released the sleeve gastrectomy can be constructed over a 36 Fr bougie (Figure 4B). The gastrectomy begins 3-5 cm away from the pylorus. The sequence of the first firings was black, green and golden cartridges ending with the blue ones. Total numbers of the cartridges were in between five and six. The surgeons insert the first 3-4 firings by the right trocar and the others by the left one. The assistant has to extend the stomach from the greater curvature in order to avoid torsion of the sleeve axis. During the stapling it is very important carefully to check the amount of the anterior and posterior part of the gastric tube avoiding an asymmetric position of the stapler line. Also a good anatomic orientation is important to avoid twisting the tube. For the last firings it is important the posterior dissection of the short splenic vessels and crura to complete mobilization of the gastric fundus. This use to be a very safe approach even in case of redundant fundus or very short vessels. All the staple line firings were reinforced with buttress material or absorbable running suture. 

#### Bowel mobilization and duodenoileal anastomosis

The vertical section of the greater omentum was done to facilitate the anastomosis. For the bowel counting and mobilization the surgeon moved to the left side and a sixth 5mm trocar can optionally be inserted in case of necessity at the left pararectal line just bellow to the umbilicus. The bowel was counted from the ileo-cecal valve to the jejunum. Counting of the bowel involved mobilization in each 5 to 5 cm or 10 to 10 cm with marked forceps. It is important that the distal bowel should be left at the right iliac fossae to avoid torsion of the bowel at the anastomosis. A total of 3 m of ileum was mobilized and prepared for an end-to-side anastomosis (Figures 4C and 4D).

To construct the anastomosis the surgeons moves back between patient legs. The anastomosis was performed by a two-layer absorbable running suture. The assistant maintained straight the anastomosis by traction of both endings of the first posterior layer. Finally, the Petersen space was closed with a running non-absorbable suture.

The final check of the anastomosis and the sleeve gastrectomy was performed by methylene blue test or endoscopy. A drain were placed in the right side of the patient left at the duodenal stump with the tip in the esophagogastric angle.

## RESULTS

A total of 67 patients were operated, most of them, 46 cases from female (68,7%), with a mean age of 43 years old (33-51). Mean BMI at the time of the surgery was 53.5 kg/m^2^ (50-63.5). All the preliminary data are resumed at Table 1.

**TABLE 1:** Patients characteristics and comorbidities

Patients	n=67	Characteristics
Female gender	n=46	68.7%
Age (years old)	44	33 to 56
BMI (Kg/m2)	53.5	50 to 63.5
Hypertension	n=33	49.2%
Type 2 DM	n=18	26.8%
Dyslipidemia	n=24	35.8%
Sleep Apnea	N=18	26.8%

Surgery took 115 minutes on average. There were no significant perioperative complications but five patients (12.8%) had any postoperative complication. There were two leaks, one at the duodenal stump and another at the esophagogastric angle. Only two patients had to be reoperated, one due to hemoperitoneum and the other to drain esophagogastric angle leakage. For the duodenal stump leak was not necessary reoperation as the leak healed just with clinical management. No complications at the duodenoileal anastomosis nor related to the right gastric artery ligation were reported. There was no mortality. Table 2 presents the data related to the surgery.

**TABLE 2:** Early outcomes of SADI-S with right gastric artery ligation

Surgical time (min)	115	80 to 180
Length of stay (days)	2.5	1 to 25
ICU recovery (cases)	5	7.5%
Morbidity/cases	5	7.5%
Mortality/cases	0	0%
Reoperation/cases	2	2.9%
Hemoperitoneum/cases	1	1.5%
Leak/cases	2	2.9%
Intra-abdominal collection/cases	3	4.4%

## DISCUSSION

Bariatric surgery is considered the most effective management for weight loss in patients with morbid obesity. It is also de most effective approach for improvement or remission of related comorbidities. Several technical options have been used as different alternatives of bariatric surgery. The surgeon's choice of technique will depend of distinct reasons: gender, BMI, meal preferences, age, presence of GERD, comorbidities, and option of the patient are some of them. Skills and training of the surgeon certainly will influence also. None of the surgical bariatric options can be considered the best one as a lot of factors can interfere in the final result. When we analyse the long-term results of bariatric surgery we may conclude that the different surgical techniques can achieve good result for around two thirds of the patients. However looking at the literature results with DS, considered modern version of biliopancreatic diversion, we can find the best results in terms of weight loss and control of related diseases. As well this technique will present the worse side effects regarding nutritional problems in a small group of patients^1^ ^,^ ^4^ ^,^ ^5^ ^,^ ^13^ ^,^ ^17^. 

Marceau *et al.* demonstrated a few years ago that duodenal switch was safe and effective for nearly all their patients, some of them with more than 15 years of follow-up. They reinforced also that complications at the long term are not so important with an adequate follow up protocol. Despite this strong evidence DS represents only 1.5% of the whole bariatric surgery worldwide^2^ ^,^ ^18^ ^,^ ^23^.

The main reasons for this lack of acceptance from the bariatric surgery community are related to technical surgical complexity, with the longest surgical time among the procedures, high cost related to operative time, long hospitalization, greater consume of disposables, risk of postoperative complications and morbidity, risk of long term side effects related to malabsorption and the good results of simpler procedures. All these arguments may be discussed with long-term data from Marceau*et al*. or Hess*et al*.and others^12^ ^,^ ^16^ ^,^ ^18^ ^,^ ^23^.

The technical complexity of the DS may be one the most important reasons about not gaining more popularity. In this way any simplification are welcome^3^ ^,^ ^14^ ^,^ ^24^. For this reason in 2007 Sánchez-Pernaut*et al.* described the single anastomosis duodenoileal bypass with sleeve gastrectomy, the SADI-S. This new bariatric procedure came for a simplification of the DS, converting from a Roux-en-Y reconstruction into an omega loop, a technically simpler operation. They demonstrated with their first paper the feasibility and simplicity of the procedure mimicking the results from standard DS^29^.

During the next years, SADI-S has been gaining popularity and some other groups all over the world added it to their bariatric surgical portfolio. Nowadays we may find several references about SADI-S in some published articles and at any national or international meeting of bariatric surgery^8^ ^,^ ^21^ ^,^ ^28^. 

Sánchez-Pernaute and his team have recently published results at five years of follow-up with a very low weigh loss failure and a quite acceptable rate of long-term complications. Some important aspect they had to change, related to the first generation technique, was the length of the common limb from 2 m to 2.5 m in order to avoid the hypoalbuminemia. They also published their results in terms of comorbidities improvement and remission, with good results. New studies involving larger number of patients, more comparative and long follow up data are necessary^8^ ^,^ ^29^ ^,^ ^30^.

The standards of the surgical technique for SADI-S were clearly described at Sánchez-Pernaute's*et al.* first paper. They also described the anastomosis. We have evolved the technique and introduced some interesting modifications^29^ ^-^ ^31^.

There are several options to perform the sleeve gastrectomy, we do not have yet some clear guidelines about the perfect sleeve. From the analysis of the literature can be concluded that a gastric tube with a bougie 36-40 Fr guidance, preserving a bit of stomach close to the esophagogastric angle, staying far from the incisura angularis, don't angulating or twisting the stomach, proceeding with the staple line reinforcement with suture or buttress material and starting 3 to 5 cm from pylorus, may be the keys to avoid complications in this procedure^11^ ^,^ ^27^. Nevertheless it is not clear if an associated to DS or SADI-S sleeve gastrectomy would be done in the same way respecting these concepts for a primary weight loss intention one. 

The extension of the common limb adopted by Sánchez-Pernaute*et al.* was 2 m in the beginning and was extended to 2.5 m in the second series of patients. Roslin et al. with a similar procedure establish 3 m as the ideal lenght^8^ ^,^ ^21^ ^,^ ^28^ ^-^ ^31^. Based in these previous experiences we decided to start with 3 m. For the duodenoileal anastomosis Sánchez-Pernaute*et al.* proposed an end-to-side hand-sewn in two layers with absorbable suture or semi-mechanical anastomosis with a linear stapler. We have adopted a hand-sewn anastomosis also in two layers with absorbable running suture. For the duodenoileal anastomosis of the DS some authors have described also the use of circular staplers. There are not comparative studies that support which works better, so it is difficult to recommend one or another, maybe the previous experience of the surgeon are the best guideline in this topic.

The major technical limitation for the DS and SADI-S seems to be the duodenal dissection and duodeno-ileal anastomosis^33^. In general have been described a minimum dissection of the duodenum to perform the duodenal section 3-4 cm away from the pylorus in order to join with the ileum. We found this procedure complex especially in the heavier patients with strong and short mesenterium, making difficult to move with the ileum up to connect with the duodenum with no tension. One of the difficulties to perform such anastomosis is to do it under the liver, eventually under some tension. Once this anastomosis is done with a free duodenum it remains tensionless. For that reason we applied the right gastric artery ligation initially transposed to bariatric surgery by Marchesini^19^. By the choice of doing the disconnection of the duodenal bulb by sectioning the right gastric and the right gastroepiploic arteries, the stomach, pylorus and duodenal bulb may be better mobilized to a more comfortable position in order to make the duodeno-ileal anastomosis simpler, easier and safer^19^.

The right gastric artery ligation at its root is a technical gesture imported from the Whipple's procedure that allows a good mobilization of the duodenal ending without compromising its blood supply. The ligation at its root guarantees that the blood supply from the lesser curvature of the stomach keeps unaltered supplying good vascularization for the new anastomosis. 

There are few studies in the literature supporting this maneuver. Marchesini et al have published in the eighties some paper about gastric blood circulation. At that time the concern was the devascularization of the stomach secondary to proximal gastric vagotomy^19^. It was learned that the stomach submucosal arterial plexus is very rich and sufficient to be maintained by just one of the arteries. There is also, the duodeno-pancreatectomy with pylorus preservation that has both right gastric and right gastroepiploic arteries ligated. On the other side, esophago-gastrectomy disconnects the stomach from the left gastric and left gastroepiploic arteries and also the short gastric vessels, allowing the stomach to reach the thoracic cavity and neck for the reconstruction of the alimentary tract keeping adequate blood supply. Marchesini demonstrated the feasibility and safety of this maneuver in more than 1000 patients with a very low morbidity rate. We implemented it from the beginning of our DS series with a good safety profile. In this study even considering some complications occurred,none of them were related to the right gastric artery ligation^19^.

We have been always closing the Petersen space in RYGB and we keep the same policy to the SADI-S procedure. Sánchez-Pernaute *et al.* argued in their first paper that it was not necessary because it was a big space with a low chance of herniation. We found some papers about internal hernias occurred in single anastomosis gastric bypass and it seems logical to expect for the same in this duodenoileal end-to-side reconstruction. Even though we agree the chance is low, as it is also low in the RYGB and the recommendation is to close it. Considering the risks related to the late diagnosis of the internal hernias involving strangulation, necrosis and high mortality, we strongly defend this closure. It is important to have clear the first description of the Petersen's hernia in 1900 was referred to an omega gastrojejunostomy reconstruction^26^.

Finally, it is important to know that SADI-S should be considered an experimental procedure. Not all the international societies have recognized it as a new surgical bariatric alternative. We do not have enough evidence about its safety and the long-term results are scarce by these days. We recognise its benefits and advantages, but we should be cautious. At least five years follow up of a reasonable group of patients would be necessary to have enough data to discuss the official approval and international recognition and acceptation of the technique.

We cannot assure which will be the role of the SADI-S in the future. We can assume that it will be a complement for the duodenal switch but we are not sure about if it will replace it or even will be considered the "switch killer". SADI-S and DS look similar, but they are different procedures at the end. Both procedures share a similar bowel length considering the sum of alimentary and common limbs. This common channel may be important at any time for the results of weight loss, comorbidities evolution, or even for both of them.

## CONCLUSION

The surgical systematization for the single anastomosis duodenoileal bypass with sleeve gastrectomy with the technical modification of ligation of the right gastric artery seems to be an easier procedure when compared with the regular steps of the standard DS, considering the necessity of just one anastomosis with no tension. It looks like a safe procedure considering this class of advanced bariatric surgery. There is no report of complication in this series related to the ligation of the right gastric artery.
